# Water sprinkling as a tool for heat abatement in farmed Iberian red deer: Effects on calf growth and behaviour

**DOI:** 10.1371/journal.pone.0249540

**Published:** 2021-04-22

**Authors:** F. J. Pérez-Barbería, I. Arroyo-González, A. J. García, M. P. Serrano, L. Gallego, T. Landete-Castillejos

**Affiliations:** Department of Agroforestry Science and Technology and Genetics, Game and Livestock Resources Unit, University of Castilla-La Mancha, IDR, IREC, Albacete, Spain; University of Agricultural Sciences, INDIA

## Abstract

Climatic models predict scenarios in which ambient temperature will continue increasing worldwide. Under these climatic conditions, fitness and animal welfare of many populations are expected to suffer, especially those that live in captive or semi-natural conditions, where opportunities of heat abatement are limited. We undertook an experimental design to assess the effect of heat abatement that water sprinkling might have on Iberian red deer calf growth and behaviour from birth to weaning (135 days). One group of ten mother-calf pairs lived on plots with water sprinkling (treatment) available during summer’s hottest time of the day, while the control group (nine mother-calf pairs) occupied plots with no available water sprinkling. Treatment and control groups were fed *ad libitum* and swapped between plots every seven days to minimise any plot effect. Body weight was monitored weekly and individual behaviour was recorded once or twice a week at mid-day. We observed that calves had showers under the sprinklers and wallowed in mud puddles. The results clearly indicated that calves of the treatment group showed a significant increase in body weight at weaning in comparison with the control group, with no differences between sexes (treatment: male = 56.5 kg, female = 50.3 kg; control: male = 50.3 kg, female = 46.5 kg). Mother weight and mother age effects were negligible on calf body weight at weaning. The heavier the mother the faster was the rate of growth of its offspring, irrespective of calf sex. The model indicated that although males grew significantly slower than female calves in the control group, males grew faster than females when exposed to the treatment. Calves of the treatment group spent less time drinking, less time in the shade, similar time eating and more time in motion than calves of the control group. There were no behavioural differences between calf sexes of treatment and control groups. The results indicate the importance of providing animals with opportunities of heat abatement in hot environments to improve animal growth and welfare in farmed Iberian red deer.

## Introduction

Climate models predict a rise in land surface temperature between 1 and 3.7°C for the period 2046–2100 [1], with direct negative consequences on livestock production, prevalence of diseases and parasites [2] and body growth [3].

Heat stress is the result of an imbalance between metabolic heat production inside the animal body and its dissipation to the surrounding physical environment [4, 5]. Heat stress has a profound effect on suppressing the endocrine and immune system, impairing growth and reproduction, especially on highly productive livestock [6]. Less effort has been given to assess the short-term effects of heat on individual traits of wild ungulates, because of the logistic problems involved in collecting this data [5, 7, 8].

A growing conscientious consumer market has driven the focus on improving animal welfare across the complete chain of production, and so to the interest of researchers and producers to minimize stress on farmed animals [9, 10]. A number of methods to reduce heat stress on animals under intensive farming systems have been developed. These are based on reducing direct sun radiation, improving air convection and providing animals with cooling opportunities, such as water sprinklers and atomizers [11, 12]. The efficiency of these methods has been measured using behavioural and metabolic proxies of animal heat stress, such as changes in behavioural activity, food intake, rectal temperature, metabolites and stress-related hormone concentrations (Bell et al., 1989 [13]; Dussault et al., 2004 [14]; López et al., 2018 [15]; Spiers et al., 2004 [16]; Correa-Calderon et al., 2004 [17]; Strickland et al., 1989 [68]; Turner et al., 1992 [11]), and assessing animal condition and production [6, 18]. In polygynous species, such as red deer [19–21], males are larger than females from birth and their sexual dimorphism in body size increases with age [3, 22, 23]. Pérez-Barbería et al. [3] hypothesised that calves of the most costly sex to produce, ie. males, are more sensitive to limitations in energy supply than female calves [19, 24, 25], and probably more affected under conditions of heat stress.

Red deer are well adapted to a variety of habitats and abiotic environmental conditions [26], as indicated by their worldwide distribution and the success of many introductions outside their native range [27, 28]. A prime habitat is Mediterranean woodland, which is characterised by hot and dry summers, making this season the limiting period for red deer population growth [3, 29, 30]. Despite deer adapting well to a variety of climates, deer suffer thermal stress under certain physical environmental conditions [3, 8, 31, 32]. Little research has been carried out on the effects of hot conditions on the production of farmed red deer in Mediterranean systems [3]. This is mainly due to the presumption that because air humidity is low, high temperatures on farmed deer should not be an issue to animal welfare. However, Pérez-Barbería and collaborators [3] demonstrated that calf growth in farmed Iberian red deer was impaired in hot summers.

Red deer is an economically important species in many European countries [33–35]. It provides income via hunting eco-tourism to economically depressed and depopulated rural areas, and a sustainable source of meat [36–39]. Spain is the country that sustains probably the largest red deer population in Europe [34, 40, 41] and the largest Iberian red deer populations are concentrated between 38 – 40° latitude, where maximum temperatures during the first few months of a calf’s life can reach 45°C [3]. Many of these populations live in large fenced estates in unsustainable high densities by means of feed supplementation during periods of scarcity of natural food resources [42], detrimental to the regeneration of Mediterranean woodland and biodiversity [43]. Less common are deer farms for the breeding of deer, constituted by artificial paddocks that almost entirely rely on feed supplementation [3].

There is a paucity of evidence in farmed red deer that water sprinkling has any positive effect on welfare and production. The aim of this study is to assess the effect that water sprinkling might have on the growth and behaviour of Iberian red deer calf. We hypothesise that water sprinkling will promote calf growth and modify the behavioural patterns of the animals. The predictions are: (1) calves that have access to water sprinkling do make use of it to reduce heat stress; (2) calves grow faster and achieve a heavier body at weaning when water sprinkling is available, especially male calves, as they are more energy demanding to produce than female calves; (3) calves that do not have access to water sprinkling develop an energy saving behavioural strategy, in an attempt to reduce heat stress and conserve energy. We discuss the effects that the results have on farmed red deer welfare, and its management under predicted scenarios of climate warming.

## Methods

### Hypotheses, study area and animals

Hypotheses, together with the behavioural activities recorded are summarised in Table 1. The animal handling activities carried out in this experiment were approved by the Animal Welfare and Ethical Review Body of the scientific establishment (Comité de Ética en Experimentación Animal CEEA, UCLM). The experiment was carried out at the University of Castilla-La Mancha (UCLM) deer farm experimental facilities (38°57’32.8"N 1°52’51.8"W, Albacete, Spain) between 6^th^ May 2019 and 18^th^ September 2019. The UCLM deer farm is a scientific facility for the study of reproduction, nutrition, antler growth and life history traits in Iberian red deer. The farm comprises indoor and outdoor facilities for breeding, handling and undertaking experimental work on deer and small ruminants. The experimental farm complied with Spanish animal welfare legislation. It was attended daily by qualified personnel and an expert veterinarian on deer (AJG) looked after the animals on a weekly basis. The study used 19 mother-offspring pairs that were drawn from the main herd. Mothers’ ages ranged between 2 and 9 years (mean = 4.2, median = 3.0, sd = 2.14, Table 2). Because of management logistics there were two non-breeding hinds, which were not considered experimental animals.

**Table 1:** Behavioural activities recorded in instantaneous and continuous visual observations, and their hypothesised function (thermal distress, energy saving, energy expenditure).

observation type	behavioural type	behaviour	function	response	recording unit	analysis unit	statistical analysis
continuous	feeding	eating	distress	↓	min	log-ratio	lm
		eating	distress	↓	n	log-ratio	lmm
	heat relief	drinking	distress	↑	n	log-ratio	lmm
		showering	distress	↑	n	log-ratio	lm
		wallowing	distress	↑	n	log-ratio	lm
instantaneous	motion—motionless	laying down	energy saving	↑	Y/N	factor	glmm
		standing up	energy saving	↑	Y/N	factor	glmm
		waking	energy exp	↓	Y/N	factor	glmm
		running	energy exp	↓	Y/N	factor	glmm
	grazing	grazing	distress	↓	Y/N	factor	glmm
	heat relief	In the shade (vs exposure to sun)	distress	↑	Y/N	factor	glmm

Expected response of the corresponding behavioural activity under a hypothetical scenario of high heat stress (head-up arrow: increase of behavioural activity; head-down arrow: decrease of behavioural activity); duration time of behaviour (min); number of behavioural events recorded (n); Y/N: present or absence of behaviour; type of unit used in the statistical analysis (analysis unit); linear regression (lm); linear mixed models (lmm); generalised linear mixed models using binomial family and logit link function (glmm). Recording unit: duration time, in minutes, of a particular behaviour (min); number of bouts (n); presence (Y) or absence (N) of a particular activity.

**Table 2:** Basic statistics on the number, age and body weight of the experimental animals. Mother body weights are those taken at the beginning of the experiment; calf body weights are those at birth.

			treatment			control	
		mother	calf female		mother	calf female	
				male			male
age (yr)	mean	4.1	—	—	4.2	—	—
	median	3	—	—	3	—	—
	sd	2.23	—	—	2.17	—	—
	min-max	2–9	—	—	2–8	—	—
	n	10	—	—	9	—	—
weight (kg)	mean	—	8.8	9.2	—	9.0	8.8
	sd	—	0.76	1.00	—	0.07	0.34
	n	—	3	7		2	7

Mother-calf pairs were split into two groups, taking special care to balance groups by mother age and body weight, and the sex, date of birth and body weight at birth of their calves (Table 2). Calf sex was male biased in both groups as a result of a dearth of female calves born that year (Table 2).

We monitored weekly body weight of calves and mothers between the first calf birth (6 May 2019) to weaning (18 Sept 2019). The mean calf age at weaning was 122.6 days (min = 111, max = 134). At each monitoring event animals were gathered in the fields and driven to a handling facility nearby, where they were weighed on a plate platform scale fitted with a motion hold sensor (± 0.01 kg). The procedure was supervised by a veterinarian specialised on deer. Body weight monitoring was part of farming activities to assess the condition and welfare of the animals, it was a non-regulated experimental license procedure.

### Behavioural activity

We carried out a literature review on behavioural energy expenditure and thermal stress to select a number of behaviour activities that could be used as proxies to quantify thermal distress in our experimental animals (Table 1).

Behavioural activity was recorded between 10^th^ July and 18^th^ September 2019 once or twice a week (number of observational days = 17). It was carried out from the top of a 2.4 m tall tower at a vantage point between the two experimental plots (see *Experimental plots and water sprinkling treatment* section below). The observations were mainly carried out by IA-G (number of behavioural records = 2193), expect for 5 days that IA-G had the additional assistance of a trained technician (n = 514). Animals were fitted with numbered and colour coded standard plastic farming collars (40 mm width), which with the aid of a pair of binoculars enabled easy identification of the animals from a distance. Air humidity was low during the experiment (daily mean = 56.7%) and within the range expected for the season and geographic area [3], which meant that air humidity has little effect on increasing the heat stress caused by air temperature and solar radiation [3]. Consequently, we focused our observations around the hottest time of the day, between 10:00–14:00 h and on days with no or very little cloud cover. Two hours before noon were included, so that a variety of behaviours could be observed, as there is little behavioural activity at peak temperature time.

Behavioural activities were recorded using a combination of instantaneous and continuous observations [44]. Instantaneous observations were carried out every 10 min and for each calf their motion activity was recorded: laying down, standing up, walking (≤ 48 m min^-1^), running (> 48 m min^-1^); grazing activity: grazing, not grazing; and exposure to sun radiation: under direct exposure or in the shade. Continuous observations were carried out between 08:30 and 12:00 h (UTC) to record the following behaviours: time spent eating at the feed bin, and number of bouts of eating at the feed bins, drinking, showering under sprinklers and wallowing (Table 1). The units of the different behaviours (duration, number of events) were appropriately transformed according to the pertinent statistical analysis (see *Statistical analysis*).

### Experimental plots and water sprinkling treatment

On 18^th^ May each group of animals were allocated to a 0.5 Ha plot. Each plot was fenced with a stock-proofed wire of 2 m height that at its base supported a corrugated metal sheet 1.2 m high. Shade was provided by the projection created by the corrugated metal sheet, two roofed bin-feeder sheds in each plot (51 m^2^) and a line of trees around one third of the perimeter of each plot. This meant that shade was available all day long, but it was reduced to approx. 200 m^2^ in each field at solar noon during the experimental period. The animals relied entirely on supplementary feed, as the amount of forage provided by the plots was negligible [45, 46]. The base diet year round was a well-balanced mixture of chopped alfalfa hay (58%), barley straw (30%) and orange pulp (12%), administered *ad libitum* (crude protein = 13%, crude fibre = 29%, metabolized energy = 8.9 MJ/kg). In calves this diet was supplemented with a commercial specific pelleted concentrate for deer (crude protein = 17%, crude fibre = 10.2%, fat = 3.2%, ash = 9.8%, www.en.canones-caza.com), which was dispensed inside an exclusion cage that precluded hind access. Feed was presented to deer on both-side access 14 m long belt feeders to minimise aggression during feeding. Animals had free access to water at all times, which was provided by two automatic drinking bowls, 4 m apart, in each plot. In order to minimise any plot effect, animal groups were shifted weekly between plots, coinciding with the day in which body weights were monitored. Each plot was fitted with an automatic sprinkler watering system, spatially distributed across the plot (30 and 37 sprinklers in each plot). Sprinklers were switched on depending on the meteorological conditions, on average every 2 days, a total of 111 h across 66 days and providing water between 60 and 360 min per day (mean = 101 min, sd = 57). The watering system was only switched on in the plot where the treatment group was allocated at that time, which meant that watering and treatment group were always associated, so the control group never had access to sprinkling water. Sprinklers created water puddles on terrain depressions and on areas of poor drainage. It seemed that there was enough availability of water puddles, as no agonistic behaviours were observed for access to them.

### Meteorological data

We used meteorological data from the Spanish Ministry of Agriculture, Food and Environment supplied by the regional SIAR service of Castilla-La Mancha (http://crea.uclm.es/siar/datmeteo/). Data were sourced from the meteorological station of Albacete (38°56’56.5"N 1°53’53.3"W), only 2 km from the UCLM experimental deer farm and located at the same altitude. We used daily records across the study period of mean, maximum and minimum air temperature (°C), mean relative air humidity (%), global solar radiation accumulated within day (MJ m^-2^) and rainfall (mm).

## Statistical analysis

### Water sprinkling on calf growth

One of the aims of the analysis was to assess the effect of water sprinkling on calf growth from birth to weaning, controlling for maternal condition (age and body weight) and calf traits (date of birth and sex). It is widely accepted that body growth fits an exponential asymptotic curve [3, 22, 23, 26], this type of curve and the interaction of its parameters with other explanatory terms can be efficiently modelled using non-linear mixed regression, implemented in the R software package nlme [47].

The non-linear mixed models evaluated an asymptotic regression function and its gradient of the following type, as described in Pérez-Barbería et al. [3],

$$f(x)=Asym+(R0-Asym)\times e^{{-e}^{lrc}\times t}$$ Eq 1

where *t* is calf age in days; *Asym* represents the horizontal asymptote; *R0* is the response at *t* = 0; and *lrc* is the natural logarithm of the rate constant. The starting values of the parameters of this function and their subsequent optimisation are detailed in Pérez-Barbería et al. [3], with the aid of the self-starting model implemented in the nonlinear least-squares regression package (nlm) of R software [48]. To improve the interpretation of the coefficients of the model, we plotted the response of interest by fixing the other explanatory variables to their mean values. Graphics were constructed in R using the ggplot2 package based on The Grammar of Graphics [49].

### Water sprinkling on calf behaviour

To assess if the water sprinkling treatment had any effect on calf behaviour, we used two types of analyses depending on the nature of the recorded behavioural information. Behaviours that were recorded using continuous observations (Table 1) were transformed into two types of log-ratio [50]. One using the accumulated duration time of eating at the feed bins across the total time of observations and calves [log_10_ (time eating / (total time other behaviours))]. The second using the accumulated number of events of behaviour *i* (where *i* were two behaviours, eating and drinking) across the total number of events that were not behaviours *i* [i.e. log_10_(eating events / (total events–eating events–drinking events)); log_10_(drinking events / (total events–eating events–drinking events))]. The reason why the response variables were pooled across the study, rather than over a shorter period of time (day or week), was to produce a number of behavioural records within animal meaningful for statistical analysis. These log-ratios were used as the response variables in separate linear regression models in which calf sex, treatment and its interaction were fitted as predictor terms. These models were implemented using the R software package lme4 [51] and lmerTest [52]. lmerTest provides p-values for models fitted using lme4 via Satterthwaite’s degrees of freedom approximation; as in linear mixed-effects models, determining the “correct” value of degrees of freedom in the estimate of the coefficients is meaningless [53, 54].

Behaviours recorded using instantaneous observations (Table 1) in which behaviour *i* was recorded as a factor with two levels, presence or absence, were treated as binomial response variables in separate generalised linear mixed models using logit as the link function. The models fitted calf and experimental plot as random effects and calf sex, treatment and its interaction as predictor terms. Generalised linear mixed modelling was implemented using the function glmer of the lme4 R package [55]. The coefficients of all linear mixed model were calculated using REML, as the estimates are more accurate than using maximum likelihood [54]. The variance explained by the linear mixed model was represented as *R*^*2*^ marginal (variance accounted for by the fixed effects; *R*^*2*^_LMM(m)_) and *R*^*2*^ conditional (variance accounted for by random and fixed effects; *R*^*2*^_LMM(c)_), following a method developed for linear mixed-effects models [54] and used in Pérez-Barbería et al. [29, 56]. Model selection was performed using Akaike (AIC) weights aided by the normalised probability of the Kullback–Leibler discrepancy ratio, which provides a measure of a model being preferred over a competing one [57]. The significance level (α) was fixed at 0.05 in all analyses. In both log-ratio and binomial response behavioural models, we plotted the estimated marginal means and their standard errors on bar plots. In the binomial models the logit estimated marginal means were transformed into the response variable unit (probability) to ease interpretability.

## Results

### Physical environment

**Fig 1:**
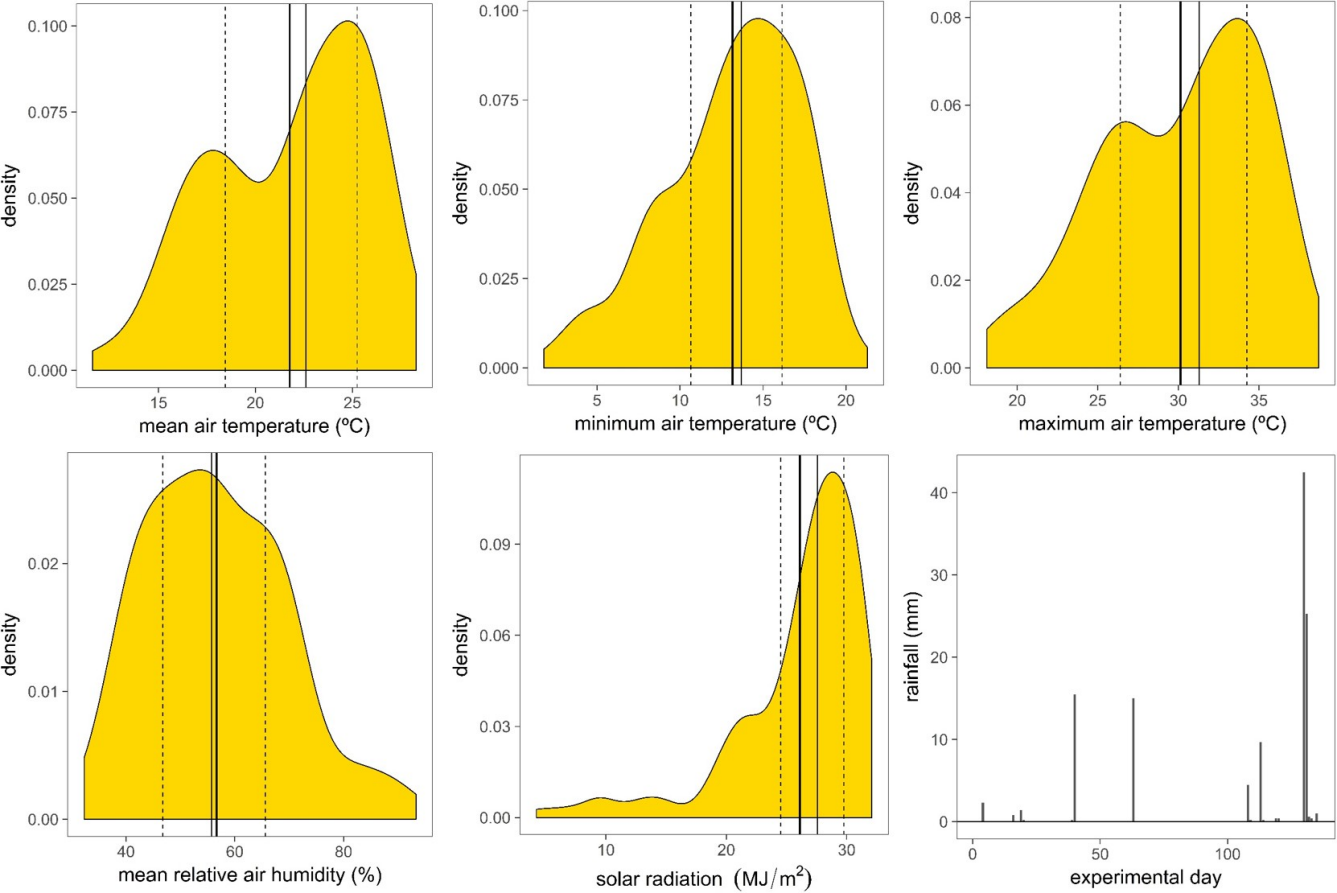
Kernel densities plots (using the Gaussian function as smoothing kernel) of means of daily mean, minimum and maximum air temperature (°C), mean relative air humidity (%) and accumulated solar radiation within day (MJ m^-2^), and daily precipitation (mm) recorded during the study period. Thick solid vertical line: mean; thin solid vertical line: median; dashed vertical lines: interquartile range. Experimental day 1 is 6^th^ of May.

During the study the daily mean of mean, mean of minimum and mean of maximum temperatures recorded were 21.8°C (Q_1_ = 18.5, Q_3_ = 25.3), 13.2°C (Q_1_ = 10.7, Q_3_ = 16.2) and 30.1°C (Q_1_ = 26.4, Q_3_ = 34.3), respectively, and the absolute minimum and maximum temperature were 1.8°C and 38.7°C (Fig 1). Mean humidity varied between Q_1_ = 46.8% and Q_3_ = 65.6%, mean = 56.7%. Mean accumulated daily solar radiation was 26.1 MJ m^-2^ (Q_1_ = 24.5 and Q_3_ = 29.8). Out of the 135 days that the experiment lasted there were 18 days with precipitation, of those only 6 days accumulated more than 4 mm. Total precipitation during the experiment was 121 mm of which 82% was accumulated in 4 days (Fig 1). Mean, minimum and maximum temperatures recorded during the time period corresponding to the behavioural monitoring of the animals (between 08:30 h and 12:00 h) were 27.7°C, 19.6°C and 35.3°C, respectively.

### Calf growth

The exponential asymptotic curve (Eq 1 Methods) used in our mixed regression models produced good fits of calf weight against calf age, as shown in S1 Fig that depicts the predictions of body weight of two calves of each sex randomly chosen against their actual body weights.

**Fig 2:**
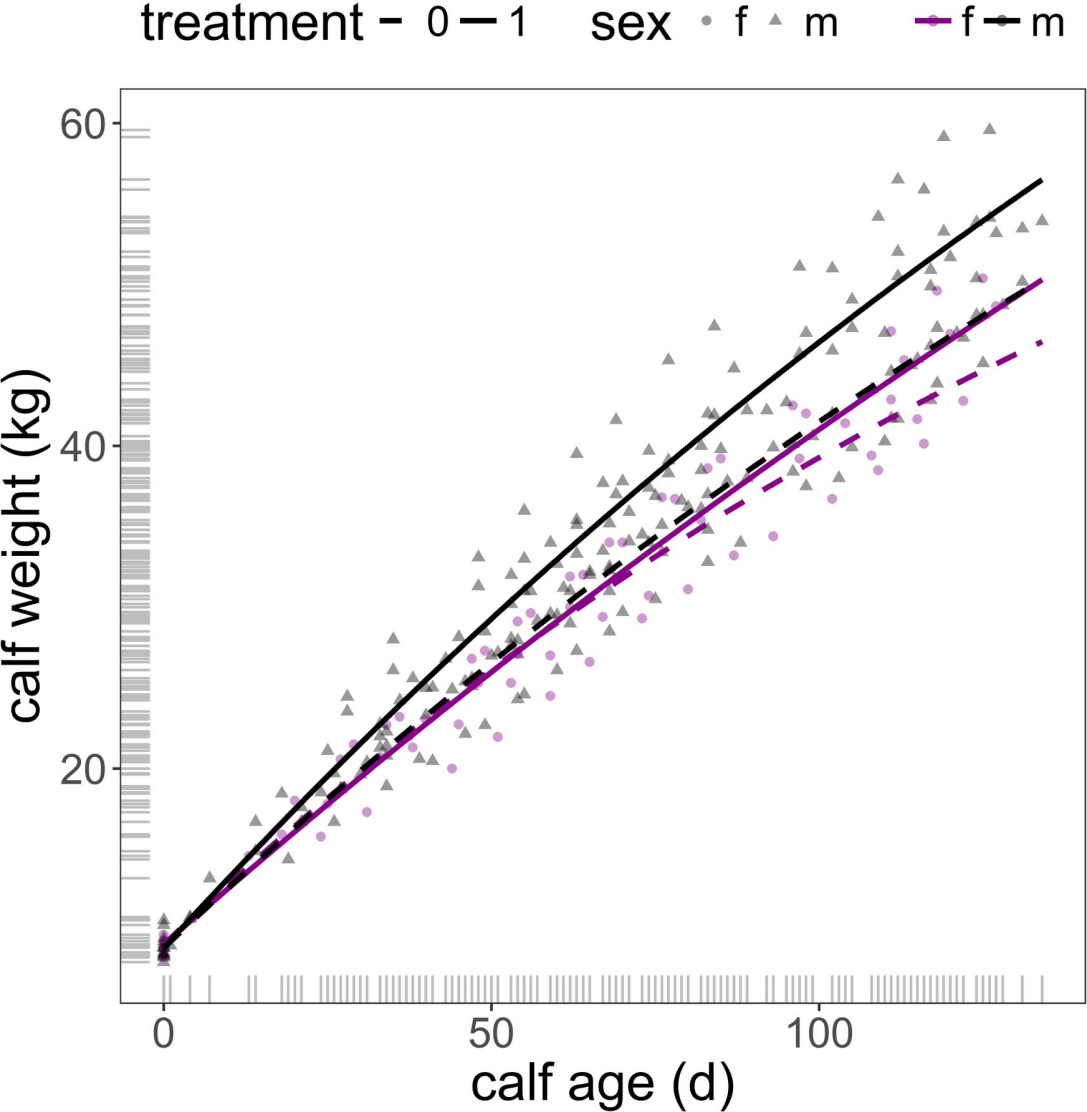
Predicted male and female calf body weight from birth (day 0) to end of experiment (day 135) from model in Table 3 (mother weight and mother age fixed at their mean values). f: female calf (magenta, circle); m: male calf (grey, triangle); treatment 1 (group with access to water sprinkling): solid line; treatment 0 control (group without access to water sprinkling): dashed line.

Interpretation of model in Table 3 requires caution, as the value of the parameters and its graphical representation in Fig 2 might seem counterintuitive. This is because (i) a feature of non-linear regression is that the parameters are strongly dependent on each other, and (ii) the asymptotes of the two non-linear curves represented by the model are out of the actual data range. Consequently, the rate of *lrc* growth parameter has to be interpreted in combination with the corresponding values of asymptotes and intercepts.

**Table 3:** Coefficients and statistics of an exponential asymptotic mixed linear model on calf weight (kg) against calf age (d) controlling for calf sex and mother weight (kg) and age (years).

Random effects	model 0	model 1	final model				
Groups (sd)							
Asym intercept calf	11.503	10.466	11.122				
Residual	0.712	0.724	0.699				
Fixed effects			estimate	se	df	t-value	*P*
Asym intercept	Y ***	Y	127.94	32.090	218	3.98	<0.001
Asym mother wt	Y **	—	-0.38	0.305	218	-1.27	0.205
Asym sex (male)	Y **	Y *	39.07	15.100	218	2.58	0.010
Asym mother age (yr)	—	Y	-1.22	2.852	218	-0.43	0.667
Asym treatment	Y *	Y *	79.43	37.528	218	2.11	0.035
Asym treatment x sex (male)	Y +	Y	-65.42	39.646	218	-1.65	0.100
R0 intercept	Y **	Y *	7.35	1.802	218	4.07	0.000
R0 mother wt	Y *	—	0.01	0.018	218	0.68	0.492
R0 sex (male)	Y	Y	-0.20	0.302	218	-0.67	0.503
R0 mother age (yr)	—	Y *	0.11	0.069	218	1.60	0.110
lrc intercept	Y ***	Y *	-6.02	0.346	218	-17.37	< 0.001
lrc mother wt	Y **	—	0.00	0.003	218	1.93	0.054
lrc sex (male)	Y **	Y *	-0.42	0.165	218	-2.59	0.010
lrc mother age (yr)	—	Y *	0.03	0.025	218	1.18	0.238
lrc treatment	Y **	Y **	-0.80	0.276	218	-2.90	0.004
lrc treatment x sex (male)	Y ***	Y *	0.83	0.299	218	2.77	0.006
AIC	671.68	678.16	666.93				
ΔAIC	4.76	11.23	0				
weights AIC	0.085	0.003	0.912				
Kullback–Leibler	0.038	0	—				

Asym: horizontal asymptote; R0: response at calf age = 0; lrc: natural logarithm of the rate constant. Y: term included in the model, Models 0, 1 and F: three competing models in the model selection process; calf ID: calf identity; df: degrees of freedom; AIC: Akaike information criterion; wAIC: AIC weights of the model; ΔAIC: delta AIC; Kullback–Leibler discrepancy ratio: normalised probability that model in column *i* is preferred over Model F; significance of the terms p-value

*** = 0–0.001

** = 0.001–0.01

* = 0.01–0.05, + = 0.05–0.1. Reference levels: female calf sex and experimental control group (i.e. no availability of water sprinkling).

Water sprinkling treatment had a significant and positive effect on the body weight of both males and females, as indicated by the predicted asymptote value of the calve’s growth of the treatment group against those of the control (treatment = 79.43, se = 37.528, p = 0.035, Table 3) and the non-significant interaction of treatment and sex on the asymptote (treatment × sex[male] = -65.43, se = 39.647, p = 0.100, Table 3, Fig 2). Maternal effects (mother weight and mother age) were negligible on the asymptotic values of the body weight of male and female calves (mother weight = -0.39, se = 0.306, p = 0.205; mother age = -1.23, se = 2.853, p = 0.667, Table 3). However, the heavier the mother the faster was the rate of growth of its offspring, this effect was marginally significant (mother weight = 0.006, se = 0.003, p = 0.055, Table 3). The model indicated that although males grew slower than females in the control group (male growth rate effect = -0.43, se = 0.166, p = 0.010) males increased their growth rate when exposed to the treatment in comparison with female calves, as indicated by the significant interaction treatment × sex (interaction effect estimate = 0.83, se = 0.300, p = 0.006, Table 3). Female calves in the treatment group grew at a lower rate than those female calves of the control group (treatment estimate = -0.80, se = 0.276, p = 0.004, Table 3, Fig 2). Calf body weight at birth did not differ between males and females (R0 sex[male] = -0.20, se = 0.302, p = 0.503; Table 3).

### Behavioural activity

**Fig 3:**
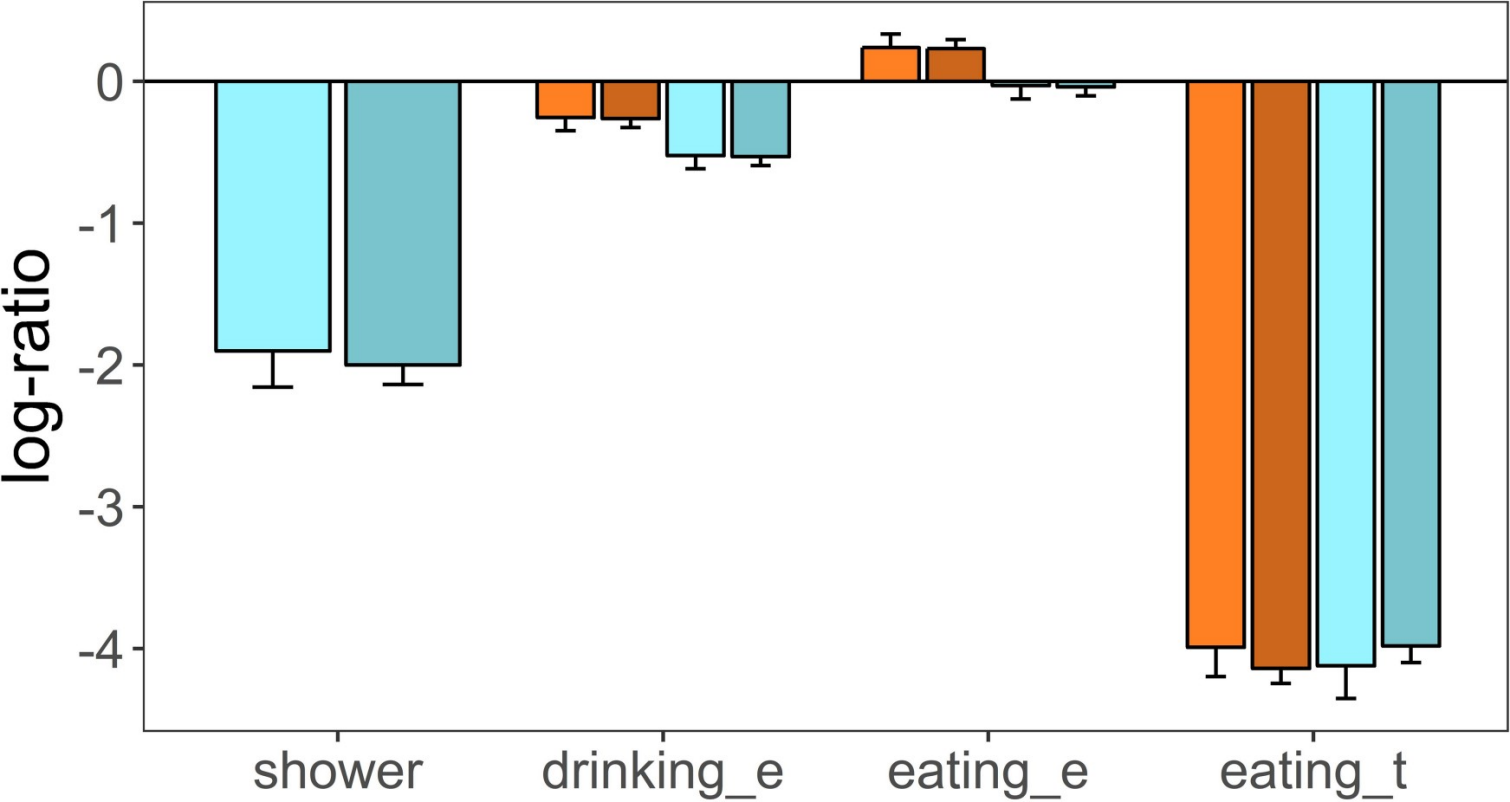
Estimated marginal means of mixed linear models on the log-ratio of different activities developed by male and female calves living in plots that differ in heat stress. Shower: deer having shower under water sprinklers; drinking_e: drinking events; eating_e: eating events; eating_t: time spent eating at feed bins; Warm colours: red deer group living in plots without access to water sprinkling heat stress abatement; Cold colours: red deer group with access to water sprinkling; Light colors: female calves; dark colours: male calves. Heat stress effect was not assessed on shower activity, as shower was only possible on the group of calves that had access to water sprinkling heat stress abatement.

There was observational evidence that calves had showers under the sprinklers and wallowed in mud puddles. Female calves spent 10.4% and 4.7%, and males 10.0% and 3.5% of their activity having showers and wallowing, respectively (total number of all activity records: 86 in females and 289 in males). The difference in the total number of activity records between calf sexes was mainly due to the larger number of males (Table 1). A linear regression analysis on the log-ratio of the number of showers plus wallowing events over all other activities, indicated that there were no significant differences between calf sexes (female estimate = -1.72, se = 0.298; male estimate = -1.84, se = 0.159, p = 0.718, Fig 3).

Calves of the control group performed more drinking events (female = 18%, male = 19%) than calves of the treatment group (female = 14%, male = 12%), and the number of drinking events between sexes was similar (Table 4). The number of eating bouts over the total number of behavioural events was similar between control and treatment groups and between sexes (control: female = 50%, male = 49%; treatment: female = 43%, male = 46%, Table 4). These results were corroborated by a linear mixed model on the log-ratio of drinking and eating events over the number of events of other activities (Table 5). The most parsimonious model among five candidate models (Table 5) was one that fitted the main effects (treatment, sex and type of behaviour [drinking, eating]) and no interaction terms (Model F). This model clearly indicated that calves of the control group visited water bowls more times than calves of the treatment group (treatment group estimate = -0.268, se = 0.077, p = 0.003, Fig 3) and this was consistent for female and male calves (sex effect: estimate = -0.008, se = 0.093, p = 0.930, Fig 3). Calves visited feed bins more frequently than water bowls, as indicated by a positive and significant eating behaviour term (estimate = 0.492, se = 0.048, p < 0.001). Treatment and sex had no effect on the number of eating events by calves, as indicated by the non-significant terms of all candidate models that included the pertinent interactions between treatment, sex and behaviour (Table 5, Fig 3). The variance accounted for by the fixed effects in Model F was 0.681 and increased to 0.819 when random effects were also fitted in the model (R^2^_LMM(m)_, R^2^_LMM(c)_, Table 5).

**Table 4:** Counts of drinking and eating events in male and female calves for control and treatment groups.

		control		treatment
	female	male	female	male
drinking	19	91	12	35
eating	53	231	37	132
others	32	146	37	122
total	104	468	86	289

Others: other activities.

**Table 5:** Coefficients of regression linear mixed models on the log-ratio of drinking and eating over all other behavioural events in red deer calves.

random effects	model 0	model 1	model 2	model 3	model F				
groups (sd)									
ID (intercept)	0.132	0.134	0.127	0.129	0.127				
residual	0.150	0.147	0.147	0.143	0.146				
fixed effects	terms in the model				estimate	se	df	t-value	*p*
intercept	Y	Y	Y +	Y +	-0.253	0.094	17.161	-2.695	0.015
treatment	Y	Y +	Y **	Y **	-0.268	0.077	15	-3.464	0.003
sex (male)	Y	Y	Y	Y	-0.008	0.093	15	-0.088	0.930
behaviour (eat)	Y **	Y *	Y **	Y ***	0.492	0.048	17	10.095	<0.001
treatment x sex	Y	Y			—	—	—	—	—
treatment x behaviour	Y	Y	Y	Y	—	—	—	—	—
sex x behaviour	Y	Y	Y		—	—	—	—	—
treatment x sex x behaviour	Y				—	—	—	—	—
R^2^_GLMM(m)_	0.671	0.673	0.684	0.687	0.681				
R^2^_GLMM(c)_	0.815	0.822	0.819	0.828	0.819				
AIC	17.098	14.431	10.946	6.653	3.547				
ΔAIC	13.551	10.884	7.399	3.105	0				
wAIC	0.000	0.003	0.019	0.170	0.805				
Kullback–Leibler	0.791	0.850	0.895	0.825	—				

Reference levels are sex = female, control experimental group (i.e. no availability of water sprinkling) and behaviour = drinking. Models 0, 1, 2, 3 and F: five competing models in the model selection process. R^2^_LMM(m)_ variance accounted for by the fixed effects; R^2^_LMM(c)_ variance accounted for by the random and fixed effects. Reference levels: females calf sex, experimental control group (i.e. no availability of water sprinkling), and drinking. AIC: Akaike information criterion; AICw: AIC weights; ΔAIC: delta AIC; Kullback–Leibler discrepancy ratio: normalised probability that model in column *i+1* is preferred over model in column *i*; Other acronyms and details in Table 3.

**Fig 4:**
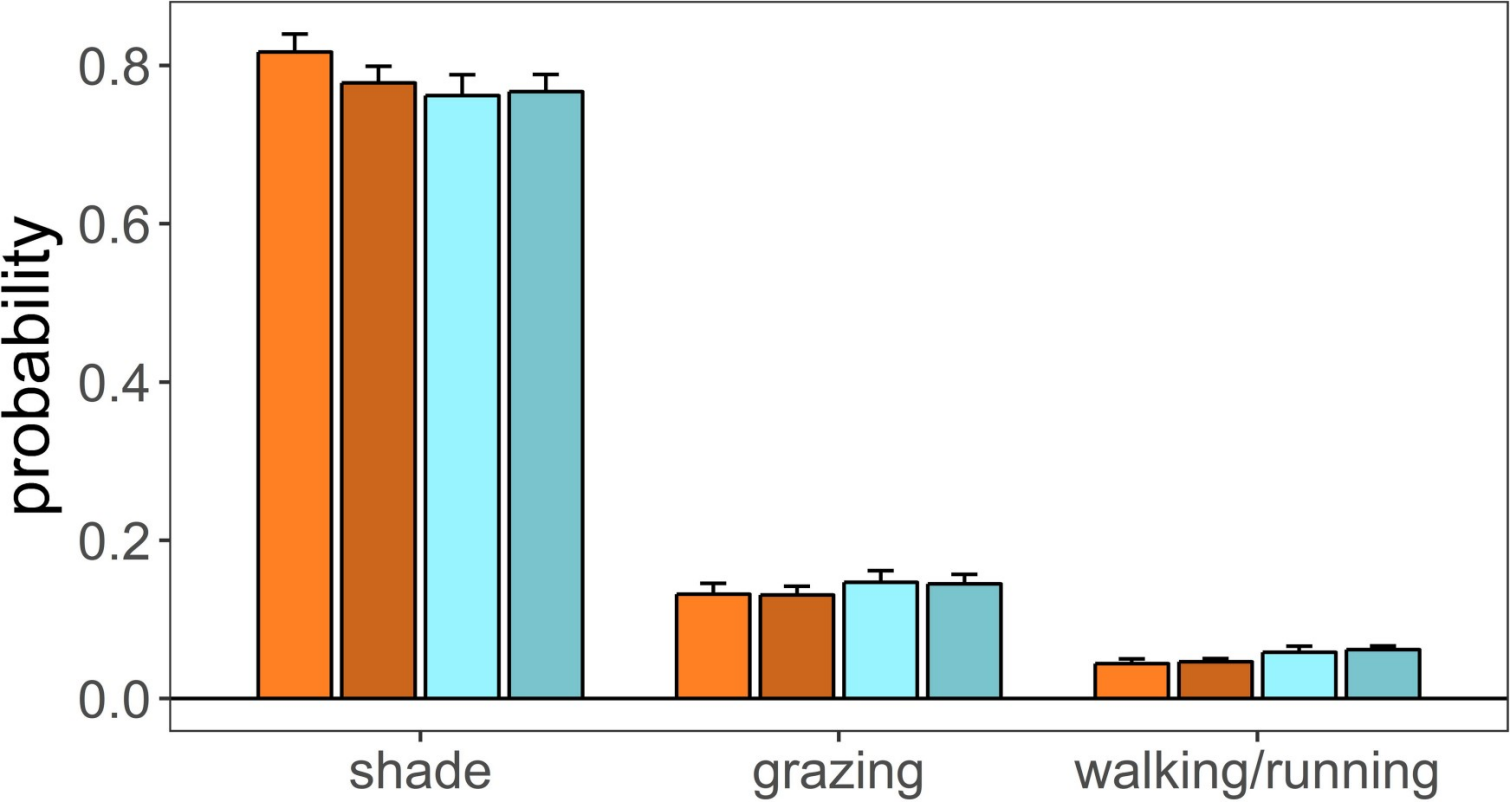
Estimated marginal means of mixed linear models on the number of events dedicated to different activities developed by male and female calves living in plots that differ in heat stress. Shade: deer observed under shade; grazing: deer grazing; walking/running: deer observed walking or running. Colour code as in Fig 3.

Time spent eating at the feed bins corroborated the results obtained above when using eating events as the response variable. Female and male calves of control and treatment groups spent a similar amount of time at the feed bins (control: female = 1.4%, male = 1.3%; treatment: female = 1.3%, male = 1.5%). A mixed linear model on the log-ratio of time spent eating at the feed bins over the total time spent at other behaviours indicated that there were no significant differences between sexes (sex [male] estimate = -0.152, se = 0.232, p = 0.512, Fig 3), between control and treatment groups (treatment estimate = -0.126, se = 0.311, p = 0.686, Fig 3) neither an interaction between treatment and sex (sex [male] x treatment = 0.295, se = 0.349, p = 0.399, Fig 3). Similarly, the time calves spent grazing did not differ between control and treatment group (treatment estimate = 0.121, se = 0.077, p = 0.116, Fig 4) neither between sexes (sex [male] estimate = -0.012, se = 0.092, p = 0.894, Fig 4).

Female and male calves of the treatment group made less use of shade than calves of the control group (treatment estimate = -0.331, se = 0.138, p = 0.017, Table 6, Fig 4). Male calves used shade less than female calves (sex [male] = -0.236, se = 0.113, p = 0.038, Table 6, Fig 4), although this seem to be a behaviour more pronounced in males of the control group (sex [male] x treatment = 0.264, se = 0.156, p = 0.090, Table 6). Female and male calves of the treatment group spend more time in motion (walking or running) than motionless (laying down or standing up) (treatment estimate = 0.300, se = 0.119, p = 0.011, Table 7, Fig 4).

**Table 6:** Coefficients of a generalised linear mixed model on the use of shade by female and male calves (binomial response and logit as the link function).

random effects	model 0	model F			
groups (sd)					
ID (intercept)	4.53E-09	6.73E-05			
fixed effects	terms in the model	estimate	se	t-value	p
intercept	Y ***	1.492	0.150	9.921	<0.001
treatment	Y +	-0.331	0.138	-2.385	0.017
sex (male)	Y	-0.236	0.113	-2.075	0.038
treatment x sex (male)	—	0.264	0.156	1.694	0.090
R^2^_LMM(m)_	0.005	0.008			
R^2^_LMM(c)_	0.030	0.032			
AIC	6090.9	6090.1			
ΔAIC	0.821	0			
wAIC	0.398	0.601			
Kullback–Leibler	0.398	—			

Models 0 and F: two competing models in the model selection process. Kullback–Leibler discrepancy ratio: normalised probability that model F is preferred over model 0; Other acronyms and details in Tables 3 and 5.

**Table 7:** Coefficients of a generalised linear mixed model on locomotion (walk, run) vs motionless (lie down, stand up) activity of female and male calves (binomial response and logit as the link function).

random effects	model 0	model F			
groups (sd)					
ID (intercept)	0	0			
plot (intercept)	0	0			
fixed effects	terms in the model	estimate	se	t-value	p
intercept	Y ***	-3.076	0.144	-21.361	<0.001
treatment	Y +	0.300	0.118	2.532	0.011
sex (male)	Y	0.054	0.143	0.378	0.705
treatment x sex (male)	Y	—	—	—	—
R^2^_LMM(m)_	0.025	0.022			
R^2^_LMM(c)_	0.025	0.022			
AIC	2392.8	2391.5			
ΔAIC	1.263	0			
wAIC	0.347	0.652			
Kullback–Leibler	0.652	—			

Models 0 and F: two competing models in the model selection process. Kullback–Leibler discrepancy ratio: normalised probability that model F is preferred over model 0; Other acronyms and details in Tables 3 and 5.

## Discussion

Our results support the hypotheses that calves living in a physical environment which offers cooling opportunities grow heavier and their behaviour is affected in agreement with theory. Thus, calves that had access to water sprinkling spent less time in the shade, drank less frequently and spent more time in motion, in comparison with calves of the control group (Table 1). Our results did not support the hypothesis that the control group was under such a level of heat stress that it impaired their food intake (Table 1), as calves of both groups and sexes spent similar time eating.

The effect of water sprinkling on weaning weight was substantial; the predicted weaning weight across female and male calves of the treatment group was 5 kg heavier (female = 50 kg, male = 56 kg) than the weaning weight of the control group (female = 46 kg, male = 50 kg). This is an increment of 10% in body weight over the predicted weaning weight of the control group (Fig 2), which corresponds to 46 g/d and 28 g/d in male and female calves over the daily growth of the control group. This is consistent with the results of Pérez-Barbería et al. [3] that found that the growth of calves of farmed Iberian red deer was impaired at high air temperatures. These authors established, in a sample of 583 calves over a period of 19 years, that at weaning (day 143) calves growing in colder summers were up to 1.2 kg heavier compared with cohorts born in hotter summers, especially male calves which are the most energy-demanding sex to produce. López et al. [15] found that calving and pre-weaning weights were lighter in cattle calves that were born under high values of a temperature-humidity index. There is evidence in farmed red deer that calves which are heavier at weaning are also heavier as adults [58]. Therefore, it seems likely that the effect that water sprinkling had on weaning weight persisted in adulthood, although it is yet to be proven.

Deer hinds in their prime reproductive stage and in good condition produce heavier offspring, especially in wild populations affected by strong variation in food supply [20, 59]. Our models on calf growth included maternal effects (mother weight and mother age) but their effects were negligible on weaning weight and marginally significant on growth rate (i.e. offspring of heavier hinds grew faster than offspring of lighter mothers after controlling for hind age). These results are not surprising given that our hinds were in good condition and that the sample size was small for detecting minor effects. Male calves grew slower than female calves, as could be explained by the fact that male adult weight is achieved by a combination of faster growth rates and a longer time growing (mule deer *Odocoileus hemionus*, [22]). As expected, male growth rate was more hampered by thermal stress than female growth rate, indicated by a significant increase in the rate of male growth vs. female growth within the treatment group. Lower rates of female growth in the treatment group did not preclude them gaining heavier weaning weights. Our results corroborate the efficacy of cooling by water sprinkling and its beneficial effect on the growth of red deer calves, as found in dairy cattle [11].

It can be argued that in our experiment calves were not only benefiting from heat abatement opportunities provided by water sprinkling but also gained from better pasture induced by water sprinkling. This is unlikely because (i) watering treatment was alternated between plots, (ii) all animals were fed *ad libitum*, (iii) pasture made a negligible contribution to the main diet, and (iv) the delay and carry over effects that watering has on vegetation growth. On this last point, watering was alternated between plots every 7 days. This means that every time the deer groups were swapped between plots, the treatment group was using a previously non-irrigated plot while the control group was using a recently irrigated one. Furthermore, even fast growing plants with basal meristems that are not easily damaged by grazing, such as grasses [60, 61], do not immediately respond to irrigation. For example, grasses grow vigorously up to levels of 40–60% of water depletion in the soil [62]. In addition, most of the energy that calves receive before weaning comes from milk, the contribution of other nutritional resources becomes significant only close to weaning [46, 63]. Furthermore, in our experiment, availability of concentrate feed meant calves close to weaning relied even less on pasture. In ruminants, mean retention time of standard forage diets is between 40–50 h (Fahey and Berger, 1988 [64]), with a peak in faecal output at 20 h in red deer [65] that declines over 7 days, which is the standard time used to carry out digestibility trials in ruminants [66]. It is therefore evident that the eventual nutritional carry-over effect of the digestion of forage on milk production in hinds clearly overlaped across the period in which water sprinkling was applied to the plots. All in all, it seems unlikely that the treatment promoted calf growth through forage or milk provisioning resources.

As expected, male and female calves of the control group made use of the shade more than calves of the treatment group, possibly as an energy saving behaviour. We also expected that males spent more time under shade than females, but actually found the opposite. This could not be explained as a consequence of males prioritising higher mobility over energy saving behaviour, compared to females, because we found no differences in mobility between sexes. However, mobility did differ between treatments; calves of both sexes being more mobile in the treatment than in the control group. Shade provisioning in farming livestock systems is one of the most common, efficient and cost-effective methods for heat abatement, even in moderately hot climates [67]. Different types of materials (fabrics, corrugated metal sheets, insulated panels) not only affect shade properties but the radiant heat reflected from these surfaces, and so influences the animal energy balance [68]. Shade from trees is preferred over shade produced by artificial materials; tree leaves block sun radiation but also cool the air by evaporating water through their stomas [4].

In an environment with air humidity of 40% and temperatures ranging between 30 and 40°C, Bell et al. [13] found that pregnant ewes reduced their food intake by 25%. There was also an increase in rectal temperature of between 0.3 and 1.0°C, compared to ewes living in thermoneutral conditions (20°C and 30% humidity). Strickland [69] found that dairy cattle living in cooled housing increased their food intake with a concomitant positive effect on milk yield and milk protein. We did not detect that water sprinkling had any effect on intake through measuring the time spent eating by calves, although we did not directly measure food intake.

It is difficult to assess the physical environmental conditions under which an animal is under thermal stress. This is mainly because a negative energy balance depends not only on the physical environment and the duration of exposure but also on animal condition and food intake. For example, Neuwirth et al. [70] monitored a number of physiological variables in cattle calves (3–4 week old) and detected heat stress only above 32.2°C at 60% air relative humidity, while other studies found significant effects on growth and behaviour under conditions of apparently less heat stress [13, 71].

Calves of the control group spent more time on low energy activities (lying or standing vs. walking or running) than did calves of the treatment group. It should be expected that animals under thermal stress minimise further increases in body temperature by restricting the use of energy-demanding activities. In sheep, energy expenditure increases by 418 J h^-1^ kg^-1^ from lying down to standing position and 2.5 J kg^-1^ from standing to walking one meter horizontally (2.0 J kg^-1^ in cattle) [66]. These energy costs are considerable, although they might seem small against estimates of daily maintenance requirements (sheep 45 kg: 6.1 MJ; cattle 500 kg: 41.8 MJ). Laporta et al. [72] used time standing as a proxy for pre-weaning activity in cattle calves whose mothers were exposed to heat stress during pregnancy; calves from mothers not exposed to heat stress spent more time standing, were heavier at birth and grew bigger than calves whose mothers were exposed to heat stress.

Water has two physical properties that make it a unique coolant for efficient body temperature regulation, (i) high specific heat, and (ii) high thermal conductivity. The first allows, via panting and sweating, large amounts of heat to be removed from the body (e.g. water latent heat binds 2,260 J per 1 g of vaporised water [4]). The second allows fast dissipation of heat from the core of the body to the environment, which is also facilitated by the fast circulation of blood and well irrigated body surface [73].

In mammals, the main routes of water acquisition are drinking, ingesting moist food, and, to a lesser extent, absorption through the skin. Ruminants increase their water turnover as ambient temperature increases. Squires [73] found a 15% increase in water intake in cattle when temperature increased from 18°C to 24°C. Also in cattle, drinking intake decreases as the moisture content of forage increases [74]. Increasing water consumption in hot environmental conditions is a common strategy to alleviate heat stress. Broucek et al. [71] found that cattle calves exposed to high temperatures maintained homeostasis by increasing water consumption but still grew less from birth to weaning than calves living in cooler environments. Our results clearly indicate that calves of the treatment group had lower water turnover than those of the control group. This suggests that animals of the control group could be losing more water than those of the treatment group via respiration and sweat, and that their water intake through the moisture of the pasture was lower than that of the treatment group.

Water sprinkling faciliates the creation of microclimates that can provide cooling oportunities to the animals which occupy that space. For example, irrigation can reduce soil surface temperature and air temperature (measured at 30 cm height) up to 6°C and 8°C, respectively [62]. This is a significant drop in temperature that the calves of our experiment could have used to mitigate thermal stress. The presence of this microclimate provides calves with cooling oportunities without the need to make direct use of the sprinklers (i.e. having showers) or wallowing, but simply by lying or standing in the areas where the plot was irrigated.

Another benefit of this physical microclimate is that it provides longer-term opportunities for cooling, in comparison with showering, which in our study was only available during a short period of the day. Red deer enjoy wallowing in the wild and on farms (www.deernz.org), as was observed in our animals. However, it is uncertain whether this is for thermo-regulation, hair removal, playing, marking territory or minimising insect harassment [75]. It has been suggested that a mudded coat protects animals from bitting and blood-sucking insects [76], but whether it has any thermal regulatiory effect is yet to be proven. A cooling effect on skin temperature occurs when water in mud evaporates from an animal’s coat. However, once the mud is dry and air gets trapped beween the skin and the resulting crust, water evaporation through the skin is impaired which reduces cooling efficiency [4]. Besides, red deer wallow in hot weather and also in cold weather across a wide range of latitude (pers. observation), which suggest that wallowing has additional functions other than cooling.

The population of biting flies in our experimental plots was very small, evidenced by the fact that the field experimenters were not stung or harrassed by insects while observing. This suggests that preventing insect harrassment was not the main cause for deer to wallow [77, 78]. The fact that deer have a natural instinct to wallow makes the provision of such opportunities advisable for welfare purposes, regardless whether wallowing has a significant effect on skin cooling. However, issues have been raised against wallowing in farming systems, such as it being a potential focus for diseases and a risk of faecal spills to nearby water bodies [75, 79]. This suggests the need for a risk assessment to wallowing.

Deer farming in Mediterranean habitats can contribute to the economy of deprived rural areas and counter the abandonment of the countryside, which is a worrying issue in many parts of Europe [80]. For an economic benefit, deer farms need be profitable and sustainable, which is often not the case in Mediterranean habitats [81]. Pérez-Barbería et al. [3] argued that current climatic data, and projections of temperature rises in Spain, indicates that some areas such as the north-west are less likely to provide environments where deer are subject to heat stress, either now or in the future. These areas have provided excellent grazing conditions to the nomadic Iberian livestock [82] for hundreds of years and are less likely to suffer droughts. Such climates and habitats should therefore be considered for the establishment of sustainable and profitable deer farming. Water is a valuable resource for Mediterranean livestock and agricultural systems, where surface and groundwater use is heavily regulated at national and regional levels [83]. Therefore, costs associated with water sprinkling as a method of heat abatement need be taken into consideration, as it has evident benefits to animal welfare.

## Conclusions

Cooling oportunities provided by water sprinkling increased weaning weight and deer The results indicate that the use of water sprinkling is a good way both to improve calf growth in farmed red deer and their welfare in hot environments. Currently, these results provide the first evidence in red deer of a response in body condition and behaviour to heat stress abatement.

It is recommended that a combination of different methods of reducing heat stress are used to increase the number of cooling opportunities, providing animals with a choice according to the environmental conditions.

Predicted scenarios of climate warming emphasise that heat stress abatement in farmed ruminants is one of the most important management issues affecting animal production and welfare.

## Supporting information

S1 Fig Predicted body weights of two females and two males against age, using a non-linear mixed model that evaluated the exponential asymptotic curve in eq. 1 (see Methods). Male: solid line; female: dashed line. Different point shapes (triangle, circle, cross, square) represent actual body weights of four random different calves.
(DOCX)
