# Supplementary material for: Water sprinkling as a tool for heat abatement in farmed Iberian red deer: Effects on calf growth and behaviour
Source: PLoS One. 2021 Apr 22;16(4):e0249540. doi: 10.1371/journal.pone.0249540 (PMC8062043; doi:10.1371/journal.pone.0249540)

# S1 Fig

S1 Fig. Predicted body weights of two females and two males against age, using a non-linear mixed model that evaluated the exponential asymptotic curve in Eq. 1 (see Methods). Male: solid line; female: dashed line. Different point shapes (triangle, circle, cross, square) represent actual body weights of four random different calves.


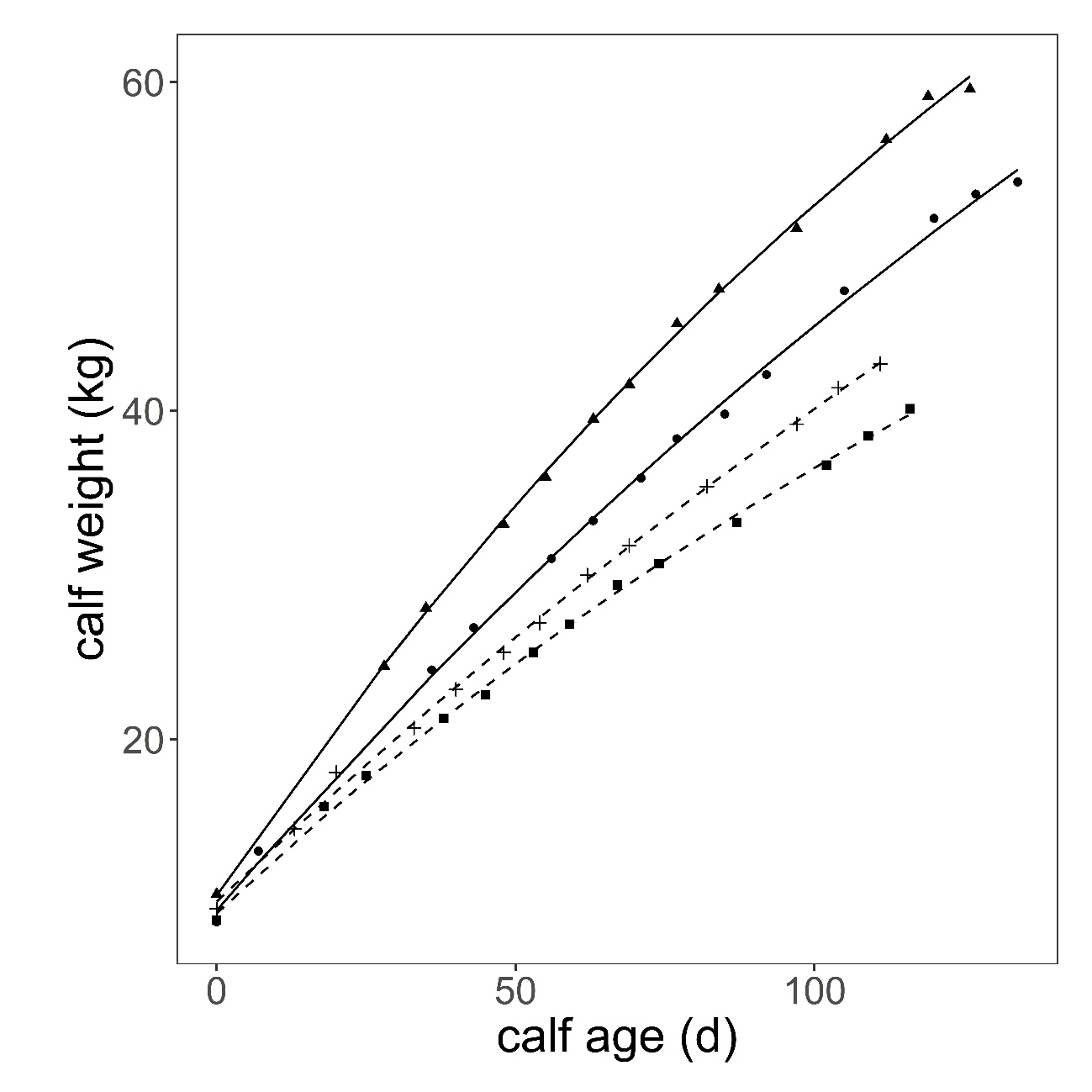

Supplement: S1 Fig — Male: solid line; female: dashed line. Different point shapes (triangle, circle, cross, square) represent actual body weights of four random different calves. (DOCX) [file pone.0249540.s001.docx]
